# Pathological personality dimensions and neurobiological emotional reactivity

**DOI:** 10.1017/S0033291724001946

**Published:** 2024-10

**Authors:** Sarah B. Barkley, Jacob Feldman, Adina Levy, Alex Grieshaber, Brady D. Nelson

**Affiliations:** 1 Department of Psychology, Stony Brook University, Psychology B Building, Stony Brook, NY, USA; 2 Department of Psychology, University of Pittsburgh, Pittsburgh, PA, USA

**Keywords:** emotions, event-related potentials, late positive potential, personality, psychopathology

## Abstract

**Background:**

The Hierarchical Taxonomy of Psychopathology (HiTOP) offers a promising framework to identify the neurobiological mechanisms of psychopathology. Many forms of psychopathology are characterized by dysfunctional emotional reactivity. The late positive potential (LPP) is an event-related potential component that provides an index of neurobiological emotional reactivity. Several categorical disorders have demonstrated a similar association with the emotion-modulated LPP. It is possible that higher-order dimensional representations of psychopathology might explain the comparable results. The present study examined the association between HiTOP-consistent pathological personality dimensions across multiple levels of the hierarchy and neurobiological emotional reactivity.

**Methods:**

The sample included 215 18–35-year-old adults (86% female) who were oversampled for psychopathology. Participants completed the emotional interrupt task while electroencephalography was recorded to examine the LPP. Participants also completed the Comprehensive Assessment of Traits relevant to Personality Disorders to assess pathological personality.

**Results:**

At the spectra level, higher negative emotionality was associated with a larger emotion-modulated LPP, while higher detachment was associated with a smaller emotion-modulated LPP. There were no associations between higher-order psychopathology levels and the emotion-modulated LPP. Compared to categorical diagnoses, spectra-level personality pathology dimensions significantly improved the prediction of the emotion-modulated LPP.

**Conclusions:**

The present study indicates that HiTOP spectra levels of negative emotionality and detachment demonstrate unique associations with neurobiological emotional reactivity. The study highlights the utility of examining dimensional and hierarchical, rather than categorical, representations of psychopathology in the attempt to identify the neurobiological origins of psychopathology.

The Hierarchical Taxonomy of Psychopathology (HiTOP) is an empirically derived model that offers a promising framework for the classification of psychopathology (Kotov et al., 2017). The dimensional and hierarchical nature of the model accounts for both higher-order commonalities and lower-order distinctions between psychopathologies, thereby addressing the long-standing issues of heterogeneity within and comorbidity between disorders using traditional categorical classifications (Kotov et al., 2021). Importantly, these framework characteristics make HiTOP-derived dimensions ideal targets to determine how neurobiological mechanisms play a role in psychopathology (Latzman, DeYoung, & HITOP Neurobiological Foundations Workgroup, 2020; Perkins, Latzman, & Patrick, 2020).

It has been a challenge to directly measure HiTOP-derived dimensions that are largely based on factor analysis. Symptom-based scales often focus on a particular spectrum or form of psychopathology and do not capture the full range of psychopathology. Personality measures can be useful in comprehensively measuring most experiences and behaviors that constitute the wide range of psychopathology. Both the five-factor model (Costa & McCrae, 1992) and alternative model of personality disorders (Krueger, Derringer, Markon, Watson, & Skodol, 2012) have demonstrated that hierarchical personality trait systems can provide a general structural framework for psychopathology (Wright et al., 2012). Notably, both normative and pathological personality are directly represented in the HiTOP framework (Kotov et al., 2021). At the spectra level, HiTOP includes five domains (i.e. internalizing, detachment, thought disorder, disinhibited externalizing, and antagonistic externalizing) which have links to the five-factor model of personality (e.g. internalizing to neuroticism, detachment to introversion). Additionally, these five domains are closely aligned with the pathological personality dimensions suggested in the alternative model of personality disorders (i.e. negative affectivity, detachment, psychoticism, disinhibition, and antagonism). Therefore, personality provides an ideal foundation for measuring HiTOP dimensions (Widiger et al., 2019). Recent research has suggested immense promise in using these HiTOP-inspired personality dimensions to better understand neurobiological mechanisms of psychopathology (Perkins et al., 2020).

Emotional reactivity is central to many forms of psychopathology (e.g. Clark & Watson, 1991; Rottenberg, Gross, & Gotlib, 2005), and there is growing research establishing the neurobiological indicators of emotional reactivity. Electroencephalography (EEG) is an ideal tool to measure neurobiological emotional reactivity due to its excellent temporal resolution, sensitivity to subtle characteristics of emotional stimuli, and ability to be measured across the lifespan. The late positive potential (LPP) is an EEG event-related potential (ERP) component hypothesized to index attentional and elaborative processing of motivationally salient information. The LPP begins 200 ms after stimulus onset (Cuthbert, Schupp, Bradley, Birbaumer, & Lang, 2000) and is maximal at centroparietal electrodes (Hajcak & Olvet, 2008). The LPP is larger in response to pleasant and unpleasant relative to neutral stimuli (Weinberg & Hajcak, 2010) – and this increased response is known as the emotion-modulated LPP.

Several studies have utilized the emotion-modulated LPP to explore emotional dysregulation in psychopathology. Across internalizing disorders, a more blunted LPP to emotional (Weinberg, Perlman, Kotov, & Hajcak, 2016) and unpleasant stimuli (Foti, Olvet, Klein, & Hajcak, 2010; MacNamara, Kotov, & Hajcak, 2016) has been associated with depression, while a more enhanced LPP to emotional (Weinberg & Sandre, 2018) and unpleasant stimuli (MacNamara et al., 2016; Moser, Huppert, Duval, & Simons, 2008) has been associated with generalized anxiety, panic, and social anxiety. The similar findings across both pleasant and unpleasant stimuli in depression are consistent with the emotion context insensitivity theory (Rottenberg et al., 2005), suggesting that depression may be related to overall attenuated emotional reactivity. Similar results across purportedly distinct disorders suggest there might be higher-order commonalities that better represent the association between neurobiological emotionality reactivity and psychopathology. Indeed, this is consistent with prominent models and theories of psychopathology. For example, a tripartite model suggests that high negative emotionality is characteristic of both depression and anxiety disorders, while low positive emotionality is more unique to depression (Clark & Watson, 1991). Interestingly, one study found that a higher-order internalizing dimension was associated with a larger emotion-modulated LPP (Rozalski & Benning, 2019), suggesting that the shared aspect between the disorders (i.e. negative emotionality) may be affiliated with enhanced emotional reactivity. In contrast, emotional dimensions related to detachment have been associated with decreased emotional reactivity. For example, low positive affect has been associated with a blunted LPP to both pleasant and unpleasant images (Weinberg & Sandre, 2018). The combination of a blunted LPP and heightened amygdala activity to unpleasant stimuli has also been shown to prospectively predict increased dysphoria (Bauer, Wilson, Phan, Shankman, & MacNamara, 2023). Moreover, a separate study found that extraversion was associated with an enhanced LPP to emotional images (Speed et al., 2015), suggesting that introversion (and its maladaptive form detachment) might be associated with a more blunted emotion-modulated LPP.

Across externalizing disorders, a more blunted LPP to unpleasant stimuli has been associated with psychopathic traits (Medina, Kirilko, & Grose-Fifer, 2016; Sadeh & Verona, 2012) and binge drinking (Connell, Patton, & McKillop, 2015), while a larger LPP to unpleasant stimuli has been associated with attention-deficit hyperactivity disorder (ADHD) (Shushakova, Ohrmann, & Pedersen, 2018). The opposite results, as well as one investigation showing that a higher-order externalizing factor was not associated with the LPP (Rozalski & Benning, 2019), suggest that it is important to distinguish between disinhibition and antagonism when examining emotion dysregulation in externalizing psychopathology. It is possible that higher-order dimensional representations of psychopathology, such as pathological personality, might explain the overlapping as well as distinct results. However, to date no study has comprehensively examined pathological personality dimensions and the emotion-modulated LPP.

The present study examined the association between pathological personality dimensions and neurobiological emotional reactivity. Importantly, the study examined these relationships across different hierarchical levels of psychopathology, including psychopathology spectra, lower-order traits, and higher-order dimensions. The final sample included 215 18–35-year-old adults who were oversampled for lifetime psychopathology and completed the emotional interrupt task while EEG was recorded to measure the LPP. Participants also completed the self-report Comprehensive Assessment of Traits relevant to Personality Disorder (CAT-PD; Simms et al., 2011) to assess negative emotionality, detachment, psychoticism, disinhibition, antagonism, and anankastia spectra as well as affiliated lower-order maladaptive traits (Ringwald et al., 2023a). The CAT-PD spectra can also be combined to estimate higher-order dimensions. To assess lifetime categorical diagnoses, participants completed the Structured Clinical Interview for the DSM-5 (SCID-5). Based on the reviewed literature, we hypothesized that relationship between personality pathology and the emotion-modulated LPP would exist at the spectra level but not the lower-order maladaptive trait or higher-order dimension (e.g. internalizing, externalizing) levels. Specifically, we hypothesized that negative emotionality and disinhibition would be associated with a larger emotion-modulated LPP, while detachment and antagonism would be associated with a smaller emotion-modulated LPP. We did not make specific hypotheses about the other pathological personality domains (psychoticism and anankastia) due to the more limited literature between these dimensions and the LPP. We also hypothesized that dimensional measures of personality pathology would significantly improve the prediction of the emotion-modulated LPP compared to the use of categorical diagnoses.

## Method

### Participants

The study consisted of 249 18–35-year-olds (*M* = 23.06, s.d. = 3.82; 86% assigned sex female). Participant racial identity included 64.0% White, 9.2% Asian, 8.3% more than one race, 7.8% Black or African American, and 0.4% Native Hawaiian or Pacific Islander. Participant ethnicity included 20.6% Hispanic. Participant sexual orientation included 45.3% heterosexual, 24.9% bisexual, 9.8% pansexual, 8.4% queer, 5.8% homosexual, 4.0% questioning/unsure, 1.3% prefer not to say, and 0.4% asexual. Participant education level included 8.7% high school graduate, 60.5% partial college, 25.2% college graduate, and 5.8% graduate/professional training.

Participants were recruited from the Long Island (68.98%) and New York City (31.02%) areas via online advertisements (Instagram, Facebook, Reddit), fliers, and word of mouth. Twelve different advertisements were used that targeted the cardinal symptom of multiple forms of psychopathology (ADHD, alcohol and substance use disorders, bipolar disorders, depressive disorders, eating disorders, generalized anxiety disorder, intermittent explosive disorder, obsessive-compulsive disorder, panic disorder, posttraumatic stress disorder, schizophrenia spectrum and other psychotic disorders, and social anxiety disorder). Participants were screened using a two-step approach: first, interested participants were contacted via phone and administered the SCID-5 (First, Williams, Karg, & Spitzer, 2015) screening questions; if at least one cardinal symptom was endorsed, participants completed an online self-report questionnaire containing subscales from the Inventory of Depression and Anxiety Symptoms–Expanded Version (Watson et al., 2012), the Externalizing Spectrum Inventory–Brief Form (Patrick, Kramer, Krueger, and Markon, 2013), and the CAT-PD (Simms et al., 2011) consistent with the endorsed form of psychopathology. Participants were enrolled in the study if they had a normed *T* score ⩾65. Exclusion criteria were active auditory or visual hallucinations, autism spectrum disorder, Down syndrome, history of select neurological conditions (cerebral palsy, epilepsy, multiple sclerosis, Parkinson's disease, stroke, traumatic brain injury), head injury associated with a loss of consciousness of 60 s or more, or an inability to read or write in English. Participants were compensated $25/h. Study procedures were approved by the Stony Brook University Institutional Review Board and informed consent was obtained from all participants in the study. The authors assert that all procedures contributing to this work comply with the ethical standards of the relevant national and institutional committees on human experimentation and with the Helsinki Declaration of 1975, as revised in 2008.

### Measures

#### Comprehensive Assessment of Traits relevant to Personality Disorder

The CAT-PD is a 216-item self-report measure of traits relevant to personality disorder. The CAT-PD consists of 33 facet traits of personality pathology that are consistent with the PSY-5 structure of personality traits (Wright & Simms, 2014) and the alternative model of personality disorder in the DSM-5 (American Psychiatric Association, 2013). The traits can further be combined to measure higher-order domains consistent with the HiTOP model spectra level. Each item is rated on a 5-point Likert scale ranging from 1 (*Very Untrue of Me*) to 5 (*Very True of Me*). The CAT-PD has demonstrated good internal consistency, test–retest reliability, and convergent validity (Long, Reinhard, Sellbom, & Anderson, 2021). The present study examined six-factor analytically derived domains that were calculated by applying factor loadings to each trait and summing all values: negative emotionality, detachment, psychoticism, disinhibition, antagonism, and anankastia (Ringwald et al., 2023a). Cronbach's *α* for the domains were calculated using items from traits that loaded highest on that domain and were as follows: negative emotionality *α* = 0.87, detachment *α* = 0.76, psychoticism *α* = 0.84, disinhibition *α* = 0.91, antagonism *α* = 0.92, and anankastia *α* = 0.58.

#### Structured Clinical Interview for the DSM-5 – Research Version (SCID-5-RV)

The SCID-5-RV was administered to the participants to determine lifetime presence of categorical psychopathology. Interviews were administered by B.A./B.S., M.A., or Ph.D. level interviewers who were supervised by clinical psychologists. All interviewers were trained by watching the SCID-101 training videos, watching two–three video-recorded interviews, and co-administering two–three interviews. All diagnoses were discussed as a best-estimate consensus meeting involving clinical psychologists where a final diagnosis was determined.

### Procedure

#### Emotional interrupt task

The LPP was examined using a modified version of the emotional interrupt task (Weinberg & Hajcak, 2011), which required participants to respond to a target (left- or right-pointing arrow) presented in between the presentation of the same emotional picture. The emotional interrupt task provides advantages over passive picture-viewing, including confirmation of participant attention by only examining trials in which target response was correct. Each trial consisted of a fixation cross (800 ms), followed by a neutral, pleasant, or unpleasant picture (1000 ms), followed by either a left (<) or right (>) pointing arrow (i.e. the target; 150 ms), followed by the same picture that had preceded the target (400 ms). The intertrial interval consisted of a blank screen and ranged from 1500 to 2000 ms. The task included 120 trials (40 neutral, 40 pleasant, 40 unpleasant) presented in a random order. Pictures were selected from the International Affective Picture System (Lang, Bradley, & Cuthbert, 2008). Pleasant and unpleasant images were matched on normative arousal ratings (see online Supplemental materials). The final task included 20 neutral pictures displaying objects or scenes with people, 20 pleasant pictures displaying cute animals or babies and erotica, and 20 unpleasant pictures displaying animal or gun threat and mutilation. Each picture was presented twice during the task. Participants were instructed to respond as quickly as possible to the target (left or right arrow) by clicking the corresponding left or right mouse button.

#### EEG recoding and data processing

Continuous EEG was collected using the ActiveTwo BioSemi system (BioSemi, the Netherlands). A total of 34 electrodes (standard 32 plus FCz and Iz) were used based on the international 10/20 system plus two electrodes placed on the left and right mastoids. Electro-oculogram activity was recorded using four facial electrodes: horizontal eye movements were measured via two electrodes located approximately 1 cm outside the outer canthus of the left and right eyes; vertical eye movements and blinks were measured via two electrodes placed approximately 1 cm above and below the right eye. The EEG signal was pre-amplified at the electrode to improve the signal-to-noise ratio. The data were digitized at a 24-bit resolution with a sampling rate of 1024 Hz using a low-pass fifth-order sinc filter with a half-power cut-off of 204.8 Hz. Active electrodes were measured online with respect to a common mode sense active electrode producing a monopolar channel. Data were re-referenced offline to the average of the left and right mastoids and band-pass filtered from 0.1 and 30 Hz. Eye blink and ocular corrections were conducted using established standards (Gratton, Coles, & Donchin, 1983).

A semiautomatic procedure was employed to detect and reject artifacts. The criteria applied were a voltage step of more than 50.0 μV between sample points, a voltage difference of 300.0 μV within a trial, or a maximum voltage difference of less than 0.50 μV within 100 ms intervals. Visual data inspection was conducted to detect and reject remaining artifacts. Trials were excluded if there was an artifact, if a response was incorrect or not provided, or if target reaction time was less than 150 ms (*M* = 7.06%, s.d. = 9.80%). The average number of trials available for data analysis included 37.56 (s.d. = 4.11) for neutral images, 37.00 (s.d. = 4.03) for pleasant images, and 36.99 (s.d. = 4.19) for unpleasant images, which did not differ between conditions (*p* = 0.26).

The EEG was segmented for each trial beginning 200 ms before the pre-target picture and continuing for 1200 ms (i.e. the entire duration of the pre-target picture presentation). The baseline was 200 ms prior to picture onset. The LPP was extracted as the average activity between 300 and 1000 ms after picture onset and separate averages were computed for neutral, pleasant, and unpleasant pictures. Consistent with prior literature examining the association between the LPP and psychopathology (Bauer et al., 2023; MacNamara et al., 2016; Rozalski & Benning, 2019; Speed et al., 2015), the current study focused on the emotion-modulated LPP via the difference between emotional and neutral stimuli (i.e. pleasant-neutral, unpleasant-neutral). We used a difference score approach, which, despite poorer reliability compared to each condition alone, can be useful to isolate neural activity of interest in ERPs (Clayson, Baldwin, & Larson, 2021). The neutral average was subtracted from each emotional average to create a difference score, producing two different averages for pleasant and unpleasant emotional stimuli. Consistent with previous studies (Foti, Hajcak, & Dien, 2009; Moran, Jendrusina, & Moser, 2013; Schupp et al., 2000), the LPP was extracted at a pooling of electrodes Cz, Pz, PO3, and PO4 where the relative difference between emotional and neutral stimuli was greatest across both pleasant and unpleasant stimuli. See online Supplementary materials for LPP psychometric properties and analyses involving neutral alone and all three levels of valence (neutral, pleasant, unpleasant).

### Data analysis

Participants were excluded from data analyses if they did not complete the EEG recording (*n* = 28), had fewer than 50% usable trials (*n* = 4), or did not complete the CAT-PD (*n* = 2), resulting in a final sample of 215 participants. All analyses were completed using R Statistical Software (v4.2.1; R Core Team, 2021), R studio (v2022.07.1), and the afex package (Singmann, Bolker, Westfall, Aust, & Ben-Shachar, 2023) including functions aov_car and aov_ez. Materials from external sources related to conducting and analyzing data can be found in the References section.

To examine pathological personality dimensions and the emotion-modulated LPP, we used a mixed-measure analysis of covariance (ANCOVA), with valence (pleasant-neutral *v.* unpleasant-neutral) as a within-subject factor and CAT-PD scores as mean centered continuous covariates. CAT-PD scores were examined in three separate ways. First, to examine pathological personality domains consistent with the HiTOP model spectra level, negative emotionality, detachment, psychoticism, antagonism, disinhibition, and anankastia were included as covariates. Second, to examine lower-order maladaptive traits, we conducted a separate ANCOVA for each pathological personality domain in which the maladaptive traits that primarily loaded on the domain based on Ringwald et al. (2023a) were included as covariates. Finally, we examined higher-order dimensions that combined the different CAT-PD domains. Based on a recent meta-analysis of the structural evidence supporting the HiTOP model (Ringwald, Forbes, & Wright, 2023b), we tested multiple higher-order dimensions. The first model examined internalizing (sum of negative emotionality and detachment), psychosis (psychoticism), and externalizing (sum of antagonism, disinhibition, and anankastia) dimensions as continuous covariates. Ringwald et al. (2023b) found that detachment also loaded on the psychosis factor, therefore, an analogous model was conducted that included detachment in the psychosis rather than internalizing dimension. The second model included internalizing (sum of negative emotionality, detachment, and psychoticism) and externalizing (sum of antagonism, disinhibition, and anankastia) dimensions as continuous covariates. All domains were *z* scored prior to creating the higher-order dimensions. For each model, we examined main effects of CAT-PD variables as well as CAT-PD × valence interactions. All continuous covariates were mean centered prior to their inclusion in the ANCOVA models. Finally, sequential regression was used to compare the change in *R*^2^ when adding pathological personality variables to a model using SCID-5 categorical diagnoses to predict the emotion-modulated LPP.

## Results

### Pathological personality domains and the emotion-modulated LPP

**Figure 1.**
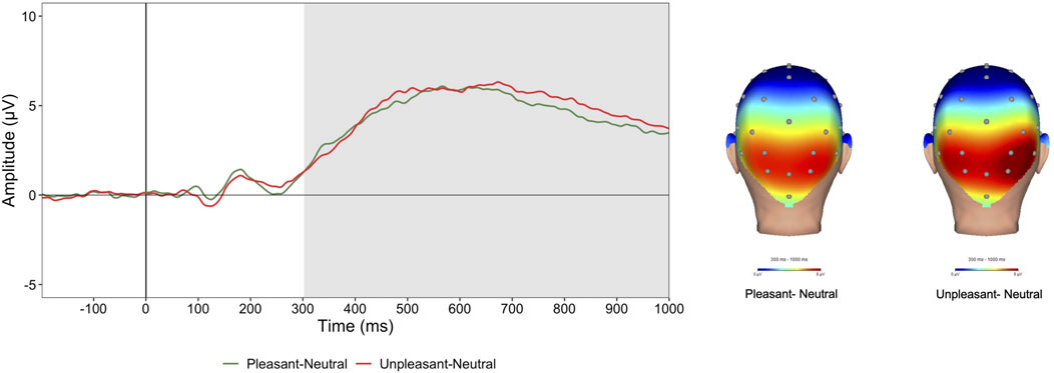
Emotion-modulated LPP waveforms at a pooling of electrodes Cz, Pz, PO3, and PO4 and scalp distributions for pleasant (pleasant-neutral) and unpleasant (unpleasant-neutral) images. The shaded region of the waveforms shows the segment from 300 to 1000 ms that was used for the scalp distributions. ms, milliseconds.

**Figure 2.**
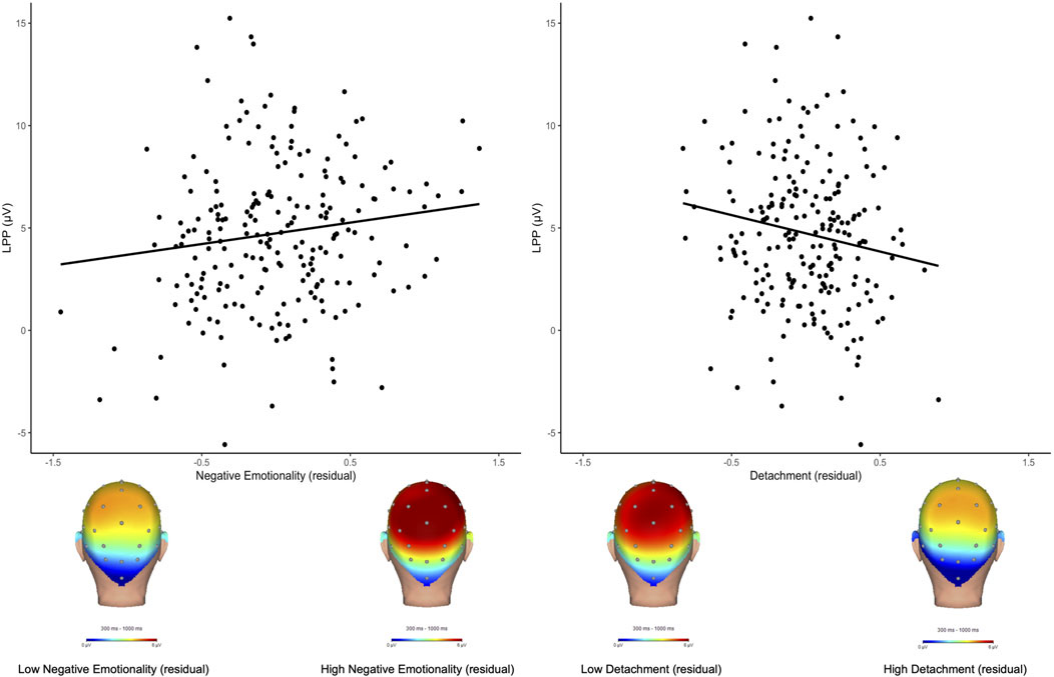
Scatterplots depict correlations between the emotion-modulated LPP and the negative emotionality residual (left) and detachment residual (right). Below depicts scalp distributions for participants with high (>1 standard deviation from the mean) and low (<1 standard deviation from the mean) negative emotionality and detachment residual scores. LPP, late positive potential.

As shown in online Supplementary materials, all CAT-PD domains were positively correlated ranging from weak (e.g. disinhibition and anankastia) to strong (e.g. negative emotionality and psychoticism). Figure 1 displays the emotion-modulated LPP waveforms and scalp distributions to pleasant and unpleasant images (see online Supplementary materials for task effects and waveforms and scalp distributions of the LPP to neutral, pleasant, and unpleasant stimuli). As shown in Table 1 and Fig. 2, analyses of CAT-PD domains and the emotion-modulated LPP indicated main effects of negative emotionality (*F*_(1,208)_ = 4.02, *p* *=* 0.046, *η*^2^ = 0.02) and detachment (*F*_(1,208)_ = 5.29, *p* *=* 0.022, *η*^2^ = 0.02), such that greater negative emotionality was associated with a larger emotion-modulated LPP, while greater detachment was associated with a smaller emotion-modulated LPP. Antagonism, disinhibition, psychoticism, and anankastia domains were not associated with the LPP. See online Supplementary materials for task behavior and picture content analyses.

**Table 1.** Pathological personality domains and the emotion-modulated late positive potential

	*F*	*p*	*η*^2^
NEG	4.02	0.046*	0.02
DET	5.29	0.022*	0.02
PSY	0.16	0.690	<0.001
DIS	0.14	0.713	<0.001
ANT	0.00	0.948	<0.001
ANA	0.02	0.880	<0.001
Valence	1.55	0.214	<0.001
NEG × valence	1.01	0.315	<0.001
DET × valence	0.08	0.774	<0.001
PSY × valence	0.28	0.598	<0.001
DIS × valence	1.87	0.172	<0.001
ANT × valence	0.65	0.421	<0.001
ANA × valence	2.57	0.111	0.01

ANA, anankastia; ANT, antagonism; DET, detachment; DIS, disinhibition; NEG, negative emotionality; PSY, psychoticism.

*Note.* This table displays *F* values, *p* values, and *η*^2^ effect sizes for pathological personality domain main effects and interactions with valence.

*indicates significance at *p* <.05.

### Maladaptive personality traits and the emotion-modulated LPP

To examine whether the domain results for negative emotionality and detachment were due to lower-order maladaptive traits, similar ANCOVA models were conducted that included either the negative emotionality or detachment traits as continuous covariates. As shown in Table 2, there were no main effects or interactions involving any of the negative emotionality or detachment maladaptive traits (all *p*s > 0.05). See online Supplementary materials for analyses involving maladaptive traits within psychoticism, antagonism, disinhibition, and anankastia domains.

**Table 2.** Maladaptive personality traits and the emotion-modulated late positive potential

	*F*	*p*
*Negative emotionality*		
Affective lability	0.00	0.97
Anger	0.61	0.44
Anxiousness	2.97	0.09
Depressiveness	1.70	0.19
Mistrust	0.51	0.48
Relationship insecurity	0.45	0.50
Health anxiety	0.12	0.73
Affective lability × valence	1.92	0.17
Anger × valence	3.00	0.08
Anxiousness × valence	0.64	0.43
Depressiveness × valence	0.09	76
Mistrust × valence	1.01	0.32
Relationship insecurity × valence	0.05	0.82
Health anxiety × valence	1.25	0.27
*Detachment*		
Anhedonia	0.01	0.90
Social withdrawal	1.85	0.18
Emotional detachment	2.79	0.10
Romantic disinterest	1.67	0.20
Anhedonia × valence	0.33	0.56
Social withdrawal × valence	0.27	0.60
Emotional detachment × valence	0.19	0.67
Romantic disinterest × valence	0.37	0.54

*Note. F* and *p* values for pathological personality traits and interactions with valence within negative emotionality and detachment domains.

### Higher-order dimensions and the emotion-modulated LPP

In the first model that examined the higher-order dimensions internalizing (negative emotionality, detachment) and externalizing (antagonism, disinhibition, anankastia), there were no main effects (internalizing: (*F*_(1,211)_ = 0.31, *p* = 0.58); externalizing: (*F*_(1,211)_ = 0.54, *p* = 0.46)) or interactions with valence (internalizing × valence: *F*_(1,211)_ = 0.50, *p* = 0.48; externalizing × valence: (*F*_(1,211)_ = 1.03, *p* = 0.32)). When detachment was included with psychosis and not internalizing, there were no main effects (psychosis: (*F*_(1,211)_ = 2.84, *p* = 0.09); externalizing (*F*_(1,211)_ = 0.49, *p* = 0.48)) or interactions with valence (psychosis × valence: (*F*_(1,211)_ = 0.01, *p* = 0.91; externalizing × valence: *F*_(1, 211)_ = 1.78, *p* = 0.18)).

In the second model that examined the higher-order dimensions internalizing (negative emotionality, detachment, psychosis) and externalizing (antagonism, disinhibition, anankastia), there were no main effects (internalizing: (*F*_(1,212)_ = 0.03, *p* = 0.85); externalizing: (*F*_(1,212)_ = 1.33, *p* = 0.25)) or interactions with valence (internalizing × valence: *F*_(1,212)_ = 0.31, *p* = 0.58; externalizing × valence: (*F*_(1,212)_ = 1.60, *p* = 0.21)).

### Pathological personality domains *v*. SCID-5 diagnoses

To compare the utility of discrete diagnoses of internalizing disorders *v.* continuous psychopathology dimensions in predicting the emotion-modulated LPP, two blocks of a linear regression model were compared using an analysis of variance. The model included the emotion-modulated LPP, averaged across pleasant and unpleasant images, as the outcome variable. The first block included SCID-5 internalizing diagnoses that have been related to the emotion-modulated LPP as predictors (major depressive disorder, generalized anxiety disorder, panic disorder with agoraphobia, social anxiety disorder, specific phobia). The second block included the same SCID-5 diagnoses in addition to the negative emotionality and detachment pathological personality domains. Compared to the first block with only diagnoses (*F*_(5,202)_ = 1.44, *p* = 0.21, adjusted *R*^2^ = 0.01), the addition of pathological personality predictors (*F*_(7,200)_ = 3.18, *p* = 0.003, adjusted *R*^2^ = 0.07) significantly improved the prediction of the emotion-modulated LPP (*F*_(2,200)_ = 7.33, *p* = 0.001). See online Supplementary materials for the results of each block and the distribution of SCID-5 lifetime diagnoses across the sample.

## Discussion

The present study indicated that the pathological personality domains’ negative emotionality and detachment were associated with a larger and smaller, respectively, emotion-modulated LPP. These associations were present irrespective of the stimulus valence (pleasant or unpleasant). Associations between personality pathology and the emotion-modulated LPP were not present at lower- or higher-order levels. Overall, the present study suggests that pathological personality dimensions consistent with the HiTOP model spectra level demonstrate unique associations with neurobiological emotional reactivity. These personality pathology domains improved the prediction of the emotion-modulated LPP compared to the use of categorical diagnoses, further emphasizing the utility of dimensional measures in clinical neuroscience.

The present study provides further clarity on the neurobiological emotional dysfunction that has been observed in purportedly distinct categorical disorders, such as anxiety disorders and depression, which at times show contrasting relationships with the LPP (MacNamara et al., 2016). The present study suggests that the association between many anxiety disorders and a larger emotion-modulated LPP could be due to elevated levels of negative emotionality. As negative emotionality has also been theorized to be a shared factor between anxiety and depression (Clark & Watson, 1991), these findings support the notion found in previous literature (Rozalski & Benning, 2019) that a higher-order internalizing factor is associated with an elevated emotion-modulated LPP. In addition to capturing commonalities between disorders, the use of HiTOP-consistent personality domains can shed light on unique aspects of psychopathology within disorders. For example, depression is characterized by both high negative emotionality and low positive emotionality/high detachment (Clark & Watson, 1991; Krueger & Markon, 2014). The present study suggests that the blunted emotion-modulated LPP often observed in depression could be the result of high detachment. Overall, these results indicate that the higher-order negative emotionality dimension might explain common relationships in the literature between some internalizing disorders and neurobiological emotional reactivity. At the same time, the results also suggest that the correlated negative emotionality and detachment dimensions can demonstrate discriminant relationships with neurobiological emotional reactivity – which likely contributes to the pervasive presence of heterogeneity within categorical disorders that involve multiple dimensions.

Overall, the CAT-PD domain findings suggest that the emotion-modulated LPP shows distinct relationships with psychopathology at the spectra level of the HiTOP model, as these domains are most consistent with the pathological personality domains used in the current study (Widiger et al., 2019). The lack of finding at the trait level suggests that differences in neurobiological emotional reactivity are not driven by one specific trait, but rather, a combination of maladaptive traits that contribute to domain-level pathology. At the same time, the lack of findings at higher-order levels suggests that it may be important to consider distinct aspects of internalizing psychopathology when examining differences in emotional reactivity. As each of the pathological personality domains are implicated in several categorical disorders, their unique relationships with emotional reactivity support the notion that a dimensional framework may be better suited to map psychopathology to neurobiology (Tiego et al., 2023). This notion is supported by our findings that the use of dimensional psychopathology variables resulted in a significant improvement in the prediction of the emotion-modulated LPP compared to the use of categorical diagnoses. Notably, categorical diagnoses did not show any associations with the emotion-modulated LPP, likely due to their heterogeneity and the high presence of comorbidity in the sample. By utilizing empirically derived, dimensional representations of psychopathology as the foundation for clinical neuroscience studies, researchers can better identify the pattern of dysregulated neurobiological reactivity among individuals with heterogeneous symptoms and comorbid disorders.

The current study did not find psychoticism or externalizing psychopathology to be associated with the emotion-modulated LPP. It should be noted that the current sample was largely characterized by females experiencing internalizing psychopathology, which might contribute to the lack of results for these types of psychopathologies. In addition, these domains might demonstrate associations with the LPP in response to disorder-specific stimuli (e.g. substance-related cues; Kroczek et al., 2018). Additional research is needed in samples with greater variability in psychoticism and externalizing psychopathology and a wider range of emotional stimuli.

The present study contained multiple strengths. Most studies examining the emotion-modulated LPP did not match pleasant and unpleasant stimuli on arousal levels. Indeed, previous studies often found a larger LPP to unpleasant relative to pleasant images, but unpleasant images were also higher in arousal levels (Weinberg & Hajcak, 2010). As the current study found associations between psychopathology and the LPP irrespective of valence, it may be important to control for arousal when examining the emotion-modulated LPP. Additionally, the present study oversampled for lifetime psychopathology, providing a more comprehensive assessment of related pathological personality dimensions. The current study also had several limitations; most notably, the gender composition of the sample was majority female (86%), which limits generalizability and investigation of sex-based differences. The sample was also mostly recruited from an area in which 82.8% of people identify as White (U.S. Census Bureau n.d.). While the current study expanded recruitment efforts and successfully increased racial diversity in our sample, certain minority groups are still underrepresented compared to the greater New York City area. In addition, the reliability of anankastia as indicated by the traits primarily loaded on the dimension was low (*α* = 0.58); however, the true reliability of the dimension was likely higher as domain scores were based on a weighting of all traits. Lastly, the cross-sectional design limits interpretation of the directionality of the findings. Future literature examining longitudinal associations between the emotion-modulated LPP and the development of pathological personality may further elucidate temporal relationships.

## Supporting information

Barkley et al. supplementary material Barkley et al. supplementary material

## Supplementary material

The supplementary material for this article can be found at https://doi.org/10.1017/S0033291724001946
