# Supplementary material for: Pathological personality dimensions and neurobiological emotional reactivity
Source: Psychol Med. 2024 Oct 15;54(13):3707–14. doi: 10.1017/S0033291724001946 (PMC11536104; doi:10.1017/S0033291724001946)
Supplement: Barkley et al. supplementary material [file S0033291724001946sup001.docx]

**Supplemental Materials**

**Image Ratings**

International Affective Picture System (IAPS; Lang et al., 2008) images included neutral (7175, 7010, 7004, 7031, 7020, 7110 , 7950, 7080, 7006, 7000, 7490, 7217, 5130, 5800, 7060, 5740, 7090, 7150, 5020, 7705, 7035, 7040, 7025, 7059, 7050, 7233, 7224, 5510, 7235, 5530, 5390, 7100, 7140, 5520, 7053, 7234, 7161, 7030, 7012, 7009), pleasant cute (2332, 2154, 2070, 2091, 1540, 2050, 1440, 1999, 1463, 1590, 2340, 2158, 1811, 1722, 2075, 1710, 2345, 2045, 2303, 2347), pleasant erotic (4232, 4672, 4651, 4607, 4656, 4694, 4608, 4687, 4693, 4660, 4695, 4683, 4652, 4810, 4311, 4681, 4670, 4668, 4220, 4290), unpleasant threat (6241, 6610, 6562, 1220, 1026, 6190, 6244, 6555, 1303, 1301, 1205, 6200, 1101, 1090, 6410, 6211, 1051, 1110, 6231, 1120), and unpleasant mutilation (3061, 3017, 3051, 3062, 3016, 9433, 3015, 3225, 3168, 9405, 3063, 3064, 3059, 3100, 3150, 3102, 3131, 3110, 3030, 3068). Table S1 displays the descriptive statistics for the normative valence and arousal ratings for neutral, pleasant, and unpleasant images (Lang et al., 2008). Pleasant images were rated as more pleasant than neutral and unpleasant images, and unpleasant images were rated as more unpleasant than neutral and pleasant images. Both pleasant and unpleasant images were rated as more arousing than neutral images, but the pleasant and unpleasant images were similar in arousal ratings.

**Split-Half Reliability**

Table S2 displays the split-half reliability of the LPP to neutral, pleasant, and unpleasant images and the emotion modulated LPP difference score at electrodes Cz, Pz, PO3, and PO4.

**Pathological Personality Domains and Neutral Stimuli**

A linear regression was conducted to examine the associations between pathological personality domains and the LPP to neutral images. Table S3 displays the results of these analyses. There were no significant associations between CAT-PD domains and the LPP to neutral images.

**Pathological Personality Domains and the Neutral, Pleasant, and Unpleasant LPP**

We also examined the association between pathological personality domains and the LPP with valence as a three-level factor. Specifically, we conducted a mixed-measure analysis of covariances (ANCOVAs) with picture valence (neutral, pleasant, unpleasant) as a within-subjects factor and CAT-PD domain scores as continuous covariates. All continuous covariates were mean centered prior to their inclusion in the ANCOVA models. Results indicated a trending valence x negative emotionality interaction (*F* (2, 416) = 2.80, *p* = .06) and a significant valence x detachment interaction (*F* (2, 416) = 3.19, *p* = .04). To follow up the interactions, we examined the correlation between the negative emotionality and detachment residuals with the LPP for each level of valence (neutral, pleasant, unpleasant). Results indicated that negative emotionality was not associated with neutral (*r*= -.06, *p*= .39), pleasant (*r*= .07, *p*= .28), or unpleasant (*r*= .02, *p*= .72) stimuli. Detachment was not associated with neutral stimuli (*r*= -.02, *p*= .72), but was negatively associated with both pleasant (*r*= -.14, *p*= .046) and unpleasant (*r*= -.15, *p*= .03) stimuli.

**Pathological Personality Descriptive Statistics and Correlations**

Table S4 displays descriptive statistics and bivariate correlations between pathological personality domains (negative emotionality, detachment, psychoticism, antagonism, and anankastia) and the emotion modulated LPP.

**Task Effects**

Figure S1 displays the LPP waveforms and three-dimensional rendered scalp distributions for neutral, pleasant, and unpleasant images. The effect of valence (neutral, pleasant, unpleasant) on the LPP was analyzed using a repeated measures analysis of variance (ANOVA). There was a main effect of valence (*F* (2, 428) = 216.40, *p* < .001, *η^2^* = 0.50). To follow up the main effect, pairwise comparisons of neutral, pleasant, and unpleasant LPPs were conducted using Tukey’s adjustment. The LPP to pleasant (*t*(214)= 16.45, *p* < .0001) and unpleasant (*t*(214)= 17.81, *p* <.0001) images was greater than the LPP to neutral images. The LPP did not differ between pleasant and unpleasant images (*t*(214)= -1.25, *p* = 0.42).

**Task Behavior**

Table S5 displays the task behavior reaction time and accuracy for the neutral, pleasant, and unpleasant image trials. There was a significant difference in reaction time across the conditions (*F* (2, 406) = 61.74, *p* = <.001), such that participants responded faster during neutral images compared to pleasant (*t*(203)= 10.03, *p* <.001) and unpleasant images (*t*(203)= 9.19, *p* <.001), which did not differ (*t*(203)= -.22, *p* = .97). There was also a significant difference in accuracy across the conditions (*F* (2, 406) = 14.17, *p* < .001), such that participants displayed increased accuracy during neutral images compared to both pleasant (*t*(203)= 4.31, *p* <.001) and unpleasant images (*t*(203)= 5.08, *p* <.001), which did not differ (*t*(203)= 0.91, *p* = .63). Follow-up tests were conducted using Tukey’s adjustment to examine pairwise comparisons of neutral, pleasant, and unpleasant images.

Pathological personality and task behavior was examined using a mixed-measure analysis of covariance (ANCOVA) with valence (pleasant-neutral vs. unpleasant-neutral) as a within-subjects factor and CAT-PD domain scores as continuous covariates. The ANCOVA was run separately for reaction time and accuracy. All continuous covariates were mean-centered prior to their inclusion in the ANCOVA models. There were no significant main effects or interactions involving any pathological personality domain (all *p*s >.10).

**Pathological Personality Domains and Picture Content**

To examine the potential relationship between picture content and pathological personality domains, we conducted two mixed-measure analysis of covariances (ANCOVAs), with picture content (cute-neutral vs. erotic-neutral or threat-neutral vs. mutilation-neutral) as a within-subjects factor and CAT-PD domain scores as continuous covariates. All continuous covariates were mean centered prior to their inclusion in the ANCOVA models. There were no significant interactions between personality pathology and picture content (all *p*s >.05).

**Pathological Personality Traits and the Emotion Modulated LPP**

Table S6 displays main effects of personality pathology traits within psychoticism, antagonism, disinhibition, and anankastia domains (Ringwald et al., 2023) as well as Valence X Trait interactions. Results indicated a main effect of workaholism within the anankastia domain, such that increased workaholism was associated with an increased emotion modulated LPP. There were no other significant main effects or interactions involving other personality pathology traits.

**Pathological Personality Domains vs. SCID-5 Diagnoses**

Table S7 displays the results of the models predicting the emotion modulated LPP (across both pleasant and unpleasant stimuli) using SCID-5 diagnoses only and when adding negative emotionality and detachment domains as predictors. In the first block, none of the SCID-5 diagnoses were associated with the emotion modulated LPP. In the second block, negative emotionality was associated with a larger LPP while detachment was associated with a smaller LPP.

**SCID-5 Lifetime Diagnoses**

Table S8 displays the distribution of SCID-5 lifetime diagnoses.

**References**

Lang, P. J., Bradley, M. M., & Cuthbert, B. N. (2008). International affective picture system (IAPS): Affective ratings of pictures and instruction manual. Technical Report A-8, Gainesville, FL: University of Florida.

Ringwald, W. R., Emery, L., Khoo, S., Clark, L. A., Kotelnikova, Y., Scalco, M. D., Watson, D., Wright, A. G. C., & Simms, L. J. (2023). Structure of pathological personality traits through the lens of the CAT-PD model. *Assessment*, *30*(7), 2276–2295. https://doi.org/10.1177/10731911221143343

**Table S1.**

*Image Valence and Arousal Ratings*

|  | Neutral | Pleasant Cute | Pleasant Erotic | Unpleasant Threat | Unpleasant Mutilation |
| --- | --- | --- | --- | --- | --- |
| Valence | 5.02 (0.44)_c_ | 7.64 (0.43)_e_ | 6.76 (0.52)_d_ | 3.59 (0.45)_b_ | 1.82 (0.31)_a_ |
| Arousal | 2.60 (0.34)_a_ | 4.94 (0.41)_b_ | 6.62 (0.27)_e_ | 5.76 (0.52)_c_ | 6.17 (0.46)_d_ |

*Note.* Higher valence values indicate more pleasant ratings and higher arousal values indicate higher arousal ratings. Subscripts indicate significant differences such that a < b < c < d < e.

**Table S2.**

*Split-Half Reliability of the LPP*

| Condition | Reliability |
| --- | --- |
| Neutral | .76 |
| Pleasant | .84 |
| Unpleasant | .77 |
| Pleasant-Neutral | .49 |
| Unpleasant-Neutral | .37 |

*Note.*  The LPP was pooled across electrodes Cz, Pz, PO3, and PO4. Reliability correlations were corrected using the Spearman-Brown Prophecy Formula for the LPP and using the Furr and Bacharach Formula for the emotion modulated LPP. LPP = late positive potential. All correlations are significant (*p* >.05)

**Table S3.**

*Pathological Personality Domains and the LPP to Neutral Images*

|  | β | *p* |
| --- | --- | --- |
| Negative Emotionality | -0.12 | .38 |
| Detachment | -0.03 | .72 |
| Psychoticism | 0.09 | .43 |
| Disinhibition | -0.22 | .09 |
| Antagonism | 0.05 | .63 |
| Anankastia | -0.02 | .79 |

*Note.* Standardized beta coefficients (β) and *p* values for the associations between pathological personality domains and the LPP to neutral images. LPP = late positive potential.

**Table S4.**

|  | 1 | 2 | 3 | 4 | 5 | 6 | 7 | 8 |
| --- | --- | --- | --- | --- | --- | --- | --- | --- |
| 1. NE |  |  |  |  |  |  |  |  |
| 1. DET | .64* |  |  |  |  |  |  |  |
| 1. PSY | .76* | .51* |  |  |  |  |  |  |
| 1. DIS | .75* | .47* | .69* |  |  |  |  |  |
| 1. ANT | .55* | .27* | .66* | .68* |  |  |  |  |
| 1. ANA | .33* | .18* | .36* | .10 | .38* |  |  |  |
| 1. LPP PLE-NEU | .13* | -.02 | .09* | .06* | .08* | .04 |  |  |
| 1. LPP UNP-NEU | .15* | -.02 | .14* | .12* | .12* | .11 | .64* |  |
| *M* | 4.13 | 1.46 | 1.34 | 2.70 | 2.87 | 1.13 | 4.59 | 4.89 |
| *SD* | 0.96 | 0.42 | 0.34 | 0.61 | 0.72 | 0.35 | 4.09 | 4.02 |

*Pathological Personality Domain Descriptive Statistics and Correlations*

*Note.* * Indicates significant correlations at *p* < .05. ANA = Anankastia; ANT = Antagonism; DET = Detachment; DIS = Disinhibition; NEG = Negative Emotionality; PSY = Psychoticism; PLE= Pleasant; UNP= Unpleasant; UNP= Neutral; LPP= Late Positive Potential; *M* = mean; *SD* = standard deviation

**Table S5.**

*Emotional Interrupt Task Behavior*

| Condition | Reaction Time  *M* (*SD*) | Accuracy  *M* (*SD*) |
| --- | --- | --- |
| Neutral | 505.55 (233.87) | 95.84 (5.75) |
| Pleasant | 536.20 (260.77) | 94.36 (6.07) |
| Unpleasant | 535.33 (261.75) | 94.23 (6.74) |

*Note.* Average reaction time (in ms) and accuracy (% correct) for task behavior during the neutral, pleasant, and unpleasant image trials. *M* = mean; ms= milliseconds; *SD* = standard deviation.

**Table S6.**

*Pathological Personality Traits and the Emotion Modulated LPP*

| **Antagonism** | *F* | *p* |
| --- | --- | --- |
| Exhibitionism | 2.29 | .13 |
| Risk Taking | 2.62 | .11 |
| Callousness | 1.19 | .28 |
| Manipulativeness | 1.04 | .31 |
| Grandiosity | 0.78 | .38 |
| Domineering | 0.28 | .60 |
| Norm Violation | 0.51 | .47 |
| Hostile Aggression | 0.00 | .97 |
| Rudeness | 0.03 | .86 |
| Rigidity | 0.53 | .47 |
| Exhibitionism X Valence | 0.43 | .51 |
| Risk Taking X Valence | 0.02 | .89 |
| Callousness X Valence | 0.22 | .64 |
| Manipulativeness X Valence | 0.20 | .66 |
| Grandiosity X Valence | 0.06 | .81 |
| Domineering X Valence | 0.11 | .74 |
| Norm Violation X Valence | 0.60 | .44 |
| Hostile Aggression X Valence | 0.00 | .99 |
| Rudeness X Valence | 1.26 | .26 |
| Rigidity X Valence | 0.08 | .78 |
| **Disinhibition** | *F* | *p* |
| Non-Planfulness | 0.64 | .44 |
| Non-Perseverance | 0.25 | .62 |
| Cognitive Problems | 0.32 | .57 |
| Fantasy Proneness | 0.51 | .48 |
| Peculiarity | 0.39 | .53 |
| Submissiveness | 1.22 | .27 |
| Non-Planfulness X Valence | 1.48 | .22 |
| Non-Perseverance X Valence | 0.78 | .38 |
| Cognitive Problems X Valence | 0.03 | .86 |
| Fantasy Proneness X Valence | 0.07 | .80 |
| Peculiarity X Valence | 0.75 | .39 |
| Submissiveness X Valence | 1.29 | .26 |
| **Psychoticism** | *F* | *p* |
| Unusual Beliefs | 0.33 | .57 |
| Unusual Experiences | 0.23 | .64 |
| Self-Harm | 0.06 | .81 |
| Unusual Beliefs X Valence | 0.73 | .40 |
| Unusual Experiences X Valence | 1.22 | .27 |
| Self-Harm X Valence | 0.00 | .99 |
| **Anankastia** | *F* | *p* |
| Irresponsibility | 2.15 | .14 |
| Perfectionism | 1.13 | .29 |
| **Workaholism** | **10.96** | **.001** |
| Irresponsibility X Valence | 0.84 | .36 |
| Perfectionism X Valence | 1.63 | .20 |
| Workaholism X Valence | 0.29 | .59 |

*Note. F* and *p* values for pathological personality traits and interactions with valence within antagonism, disinhibition, psychoticism, and anankastia domains.

**Table S7.**

*Personality Pathology Domains vs. SCID-5 Diagnoses*

| **Block 1** | β | *p* |
| --- | --- | --- |
| Major Depressive Disorder | 0.52 | .34 |
| Generalized Anxiety Disorder | -0.41 | .48 |
| Panic Disorder with Agoraphobia | 0.19 | .84 |
| Social Anxiety Disorder | 1.22 | .15 |
| Specific Phobia | -1.21 | .12 |
| **Block 2** | β | *p* |
| **Negative Emotionality** | **1.31** | **< .001** |
| **Detachment** | **-2.07** | **.007** |

*Note.* β and *p* values for block 1 including SCID-5 diagnoses and block 2 including negative emotionality and detachment personality pathology domains to predict the late positive potential.

**Table S8.**

*SCID-5 Lifetime Psychopathology Diagnoses*

|  | **Count (*n*)** | **Percent %** |
| --- | --- | --- |
| Major Depressive Disorder | 141 | 67.79 |
| Persistent Depressive Disorder | 77 | 37.02 |
| Generalized Anxiety Disorder | 60 | 28.85 |
| Post-Traumatic Stress Disorder | 82 | 39.42 |
| Eating Disorders | 70 | 33.65 |
| Panic Disorder/Agoraphobia | 21 | 10.10 |
| Specific Phobia | 29 | 13.94 |
| Social Anxiety Disorder | 23 | 11.06 |
| Obsessive-Compulsive Disorder | 18 | 8.65 |
| Bipolar Disorder | 31 | 14.90 |
| Delusions | 31 | 14.90 |
| Hallucinations | 14 | 6.73 |
| Drug Use Disorder | 61 | 29.33 |
| Alcohol Use Disorder | 54 | 25.96 |
| Attention-Deficit Hyperactivity Disorder | 49 | 23.56 |

**Figure S1.**

*LPP Waveforms and Scalp Distributions for Neutral, Pleasant, and Unpleasant Images*

**
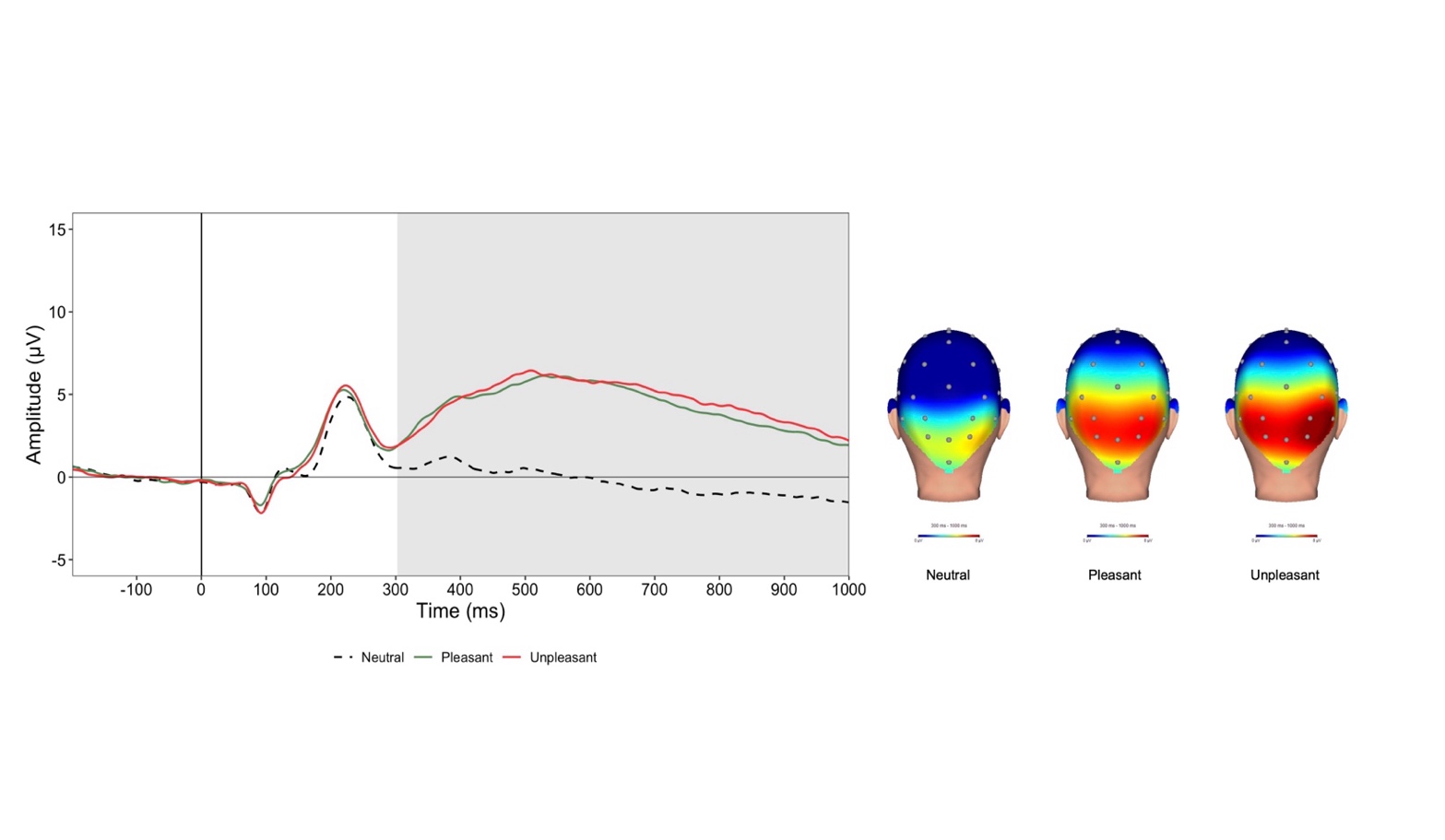
**

*Note.* Figure displays the LPP waveforms pooled at electrodes Cz, Pz, PO3, and PO4 and scalp distributions for neutral, pleasant, and unpleasant images. LPP = late positive potential. The shaded region of the waveforms shows the segment from 300 to 1000 ms that was used for the scalp distributions. ms = milliseconds
